# A critical review of bi-dimensional and three-dimensional ultrasound techniques to monitor follicle growth: do they help improving IVF outcome?

**DOI:** 10.1186/1477-7827-12-107

**Published:** 2014-11-24

**Authors:** Alberto Revelli, Giorgia Martiny, Luisa Delle Piane, Chiara Benedetto, Paolo Rinaudo, Ilan Tur-Kaspa

**Affiliations:** Gynaecology and Obstetrics, Physiopathology of Reproduction and IVF Unit, Department of Surgical Sciences, University of Torino, S. Anna Hospital, Torino, Italy; LIVET Infertility and IVF Clinic, Torino, Italy; Department of Obstetrics, Gynaecology, and Reproductive Sciences, University of California San Francisco, San Francisco, CA USA; Institute for Human Reproduction (IHR) and the Department of Obstetrics and Gynecology, University of Chicago, Chicago, IL USA

**Keywords:** Follicle size, Follicle volume, Oocyte competence, 3D ultrasound, SonoAVC, IVF outcome

## Abstract

**Background:**

This review focuses on the possibility of improving the outcome of human IVF by studying the follicles where oocytes grow by ultrasound techniques. A comprehensive analysis of bi-dimensional (2D) and three-dimensional (3D) ultrasound (US) assessment of the follicle size and volume is presented.

**Methods:**

Published reports from the year 1999 to 2014 analyzing the relationship between oocyte competence, IVF outcome and ultrasound assessment of the follicle size and volume have been critically analyzed.

**Results:**

US assessment of growing follicles has been performed mainly by 2D-US, and while overall very useful, it has been found to be of limited usefulness in predicting oocyte competence, recognize which follicles will release a mature metaphase II oocytes and decide the ideal time to trigger ovulation. In fact, a quite wide follicle size range (16–22 mm) has been reported to be associated with mature oocytes with good competence toward fertilization and embryo development. It has been also shown that smaller follicles sometimes contain mature, fertilizable oocytes. However, embryos derived from smaller follicles have probably a lower implantation potential, while follicles larger than 22 mm often contain post-mature eggs.

**Conclusions:**

The study of follicular size by 2D-US is of limited usefulness in helping in the identification of follicles containing the best oocytes and in choosing the best moment to trigger ovulation. Possibly the value of US in this area will be improved by large prospective studies in which automated 3D-US will be used.

## Background

Accurate follicular monitoring of Controlled Ovarian Hyperstimulation (COH) by transvaginal ultrasound (TV-US) is considered important for the success of human in vitro fertilization (IVF). COH, in fact, leads to the development of heterogeneous cohorts of follicles containing oocytes whose maturity and competence could be very different: it would be important to understand which follicles are developing better, with improved IVF outcomes, by using a method with low invasiveness, like TV-US.

To date, bi-dimensional (2D) US technique has been used to monitor the ovarian response and to study the growing follicles in most of the IVF cycles performed worldwide. We analyzed the available literature of the last 15 years in order to understand if 2D-US study of the follicles in which oocytes are growing is useful for: (a) the identification of follicles containing the best oocyte, and (b) the choice of the best time to give human chorionic gonadotropin (hCG) or a gonadotropin-stimulating hormone (GnRH)-agonist bolus to trigger the final oocyte maturation before ovum pick-up (OPU).

In the 2D-transvaginal scanning, the two longest diameters of each growing follicle are measured, and the mean follicle diameter is calculated. In the last years, also three-dimensional (3D) ultrasound has been tested as a tool to monitor COH during IVF, and the first data are now available.

The present review summarizes and discusses the best available evidence about how the study of follicle characteristics by US techniques may help understanding oocyte competence in order to improve IVF outcome.

## Methods

We performed a computerized MEDLINE search to identify papers published until March 2014 for the relevant studies. A combination of medical subject headings and text words were used to generate two subsets of citations, one including studies on follicular diameter in IVF and intracytoplasmic sperm injection (ICSI) (‘follicular size’, ‘follicular diameter’, ‘follicle volume’, ‘IVF’ and ‘ICSI’) and the other including studies on oocyte competence, IVF outcomes and ultrasound assessment (‘oocyte competence’, ‘developmental competence’, ‘oocyte maturation’, ‘IVF outcome’, ‘2D-US’, ‘3D-US’, ‘SonoAVC’, ‘VOCAL’). These subsets were combined using ‘AND’ to generate a set of citations relevant to the research question. In addition, cross-references of the selected studies were checked for other articles meeting the inclusion criteria, and if they were applicable, these studies were added to the pool of selected papers. No language restrictions were placed in any of the searches.

Studies were selected in a two-stage process. Firstly, two reviewers (LDP and GM) scrutinized the titles and abstracts from the electronic searches independently and full manuscripts of interest were obtained. Secondly, final inclusion or exclusion decisions were made on examination of the full manuscripts. In cases of duplicate publication, the most recent or complete versions were selected. Assessment of the manuscripts was performed independently by two reviewers (LDP and GM), and any disagreements about inclusion were resolved by consensus after consultation with a third reviewer (AR). Study characteristics such as number of oocytes involved, IVF/ICSI fertilization type, size categories, IVF outcomes, besides patient age and COH protocols for human studies and animal species for mammalian studies, were extracted from each study (Tables 1 and 2). The search for animal studies was limited only to cattle and mammalian large animals, and the exclusion criteria were the following: IVM, nuclear transfer, cloning, co-culture in cumulus cells derived from large/small follicle, parthenogenetic activation, reference only to molecular findings without clinical IVF outcomes considered.

**Table 1:** **Animal studies on follicle size and IVF outcomes**

	Animal species	Oocytes	Syze mm	% MII	% fert	% cleavage	% blastocyst formation	% hatching
Lonergan 1994 [5]	Bovines	290	6	n.e.	n.e.	n.e.	Increasing	n.e.
Feng 2007 [3]	Bovines	427	4-6	n.e.	n.e.	Increasing from 4 mm onwards	Always increasing	Always increasing
De Bem 2014 [4]	Bovines	227	3-6	n.e.	n.e.	Increasing from 6 mm onwards	Increasing from 6 mm onwards	None
Raghu 2002 [6]	Buffalos	780	3-8	n.e.	Always increasing	Always increasing	Always increasing	n.e.
Caixeta 2009 [7]	Buffalos	240	3-6-8	n.e.	n.e.	n.e.	Increasing from 8 mm onwards	n.e.
Hinrichs 2000 [8]	Horses	10753	6-11	Always increasing Together with% of condensed chromatin	n.e.	n.e.	n.e	n.e.
Khatir 2007 [9]	Dromedaries	399	6	None	n.e.	Increasing	Increasing	None, but any pregnancy under 6 mm
Crozet 1995 [2]	Goats	543	3-5	Always increasing	n.e.	Increasing from 5 mm onwards	None	n.e.
Romaguera 2011 [1]	Goats	313	3	n.e.	n.e.	None	Increasing	None
Marchal 2002 [10]	Pigs	941	3-5	Increasing from 3 mm onwards	Increasing from 3 mm onwards	n.e.	Increasing from 3 mm onwards	n.e.
Bagg 2007 [11]	Pigs	928	3-4-5	n.e.	n.e.	n.e.	Always increasing	n.e.

Only results statistically significant are reported (p < 0.05); all results are referred to size classes reported.

n.e. = outcome not evaluated.

none = any significant difference among size classes.

always increasing = increasing from each size class onwards.

**Table 2:** **Human studies on follicle size and IVF outcomes**

	Study design	Age	COH	IVF/ICSI	Oocytes	Size mm	% MII	% fert	% cleavage	% good quality embryo
Wittmack 1994 [13]	R	23-49	Long	Only IVF	6879	12-15-18-20-21-23-24	Worst recovery rate under 12 mm and over 24 mm	Increasing from 12 mm onwards	Increasing from 12 mm onwards	None
Inaudi 1995 [24]	R	28-37	Short	Only IVF	179	16-22	None in the recovery rate	n.e.	n.e.	n.e.
Dubey 1995 [26]	R	n.e.	Long	Only IVF	2429	14-16-22	n.e.	Increasing from 16 mm onwards	n.e.	n.e.
Ectors 1997 [17]	P	n.e.	Short	IVF/ICSI	2324	16-23	IVF: n.e.	IVF: increasing from 16 to 23 mm, later decreasing	IVF: increasing from 16 to 23 mm, later decreasing	IVF: increasing from 16 to 23 mm, later decreasing
							ICSI: increasing from 16 mm onwards	ICSI: none	ICSI: none	ICSI: increasing from 16 mm onwards
Bergh 1998 [18]	P	33.0	Long	IVF/ICSI	4159	16	n.e.	IVF: increasing	IVF: none	n.e. but increasing pregnancy rate only in IVF
								ICSI: none	ICSI: none	
Teisser 2000 [22]	P	28.0	Long	Only IVF	150	14	Increasing	n.e.	Increasing	n.e.
Triwitayakorn 2003 [14]	P	35.9	Long short	Only ICSI	991	10-14	Always increasing together with oocyte recovery rate	None	n.e.	none
Rosen 2008 [19]	P	34.1	Long shortantag	IVF/ICSI	2934	10-13-16-19	IVF: n.e.	IVF: always increasing	n.e.	IVF: always increasing
							ICSI: always increasing	ICSI: always increasing		ICSI: always increasing
Lee 2010 [23]	P	33.5	Long	Only ICSI	819	18-20	Increasing from 18 mm onwards	None	None	none
Mehri 2013 [20]	P	n.e.	Long	IVF/ICSI	360	15-18	Increasing from 18 mm onwards	Increasing from 18 mm onwards	None	none

Only results statistically significant are reported (p < 0.05); all results are referred to size classes reported.

n.e. = outcome not evaluated.

none = any significant difference among size classes.

always increasing = increasing from each size class onwards.

COH = controlled ovarian hyperstimulation protocol (short = short GnRH analogue, otherwise known as “flare up”; long = long GnRH analogue; antag = GnRH antagonist).

### Mammalian studies

Mammalian ovaries contain a large stock of oocytes enclosed in primordial follicles. Ovarian cyclic activity induces some of these follicles to initiate growth towards a possible ovulation. However, most of these follicles terminate their growth and degenerate through atresia. In growing follicles, only a subset of oocytes are capable to support meiosis, fertilization and early embryo development to the blastocyst stage, as shown through in vitro production experiments.

An important technical details when analyzing mammalian studies is the method of assessment of follicular growth: the majority of studies but one [1] excised follicles from animals slaughtered in commercial abattoir, and later measured the diameter with caliper or eyepiece grid. Thus the context of measuring and the consequences of puncturing follicles of different dimensions are very different from in vivo infertility clinic scenario, where an ultrasound is used.

In several species, the developmental competence of the oocyte is gained progressively during late follicular growth, after the acquisition of the competence to resume and complete meiosis. Similarly to humans, the proportion of competent oocytes in animals is positively correlated to follicular diameter. Some studies defined competence as a combination of the maturity grade of the follicle (defined by the number of cells and morphological appearance of the cumulus-oocyte complex (COC)) and follicle dimensions [2–4].

Table 1 summarizes data correlating oocyte competence to follicular size in animals [1–11].

Overall animal studies mirror human data: in follicles reaching dimensions similar to those of adult follicles, IVF outcomes are shown to be comparable. Obviously, follicle diameters and their cut-offs for maturity or postmaturity are different in different animals; but, even among the same animal species, authors reported different cut-offs, as we will later show in humans (Table 2). The age of the animals is also important: follicles excised from prepubertal animals harbor a higher proportion of small follicles with an absent/reduced oocyte developmental competence [1, 11].

### 2D-US study of follicle size and the prediction of oocyte competence

In literature there is general agreement with a better chance of retrieving an oocyte is present when a “large” follicle is punctured. The biological basis behind this concept is the following: a “large” follicle, more likely than a “small” one, may contain a free-floating COC released in the antral fluid under the influence of hyaluronic acid synthesized by cumulus cells in response to hCG [12–16]. However, there is quite a lot of confusion about the definition of ideal follicle dimension and what “large” and “small” follicle really mean. Is there a size threshold below which it is unlikely to retrieve an oocyte and/or follicles do not contain mature oocytes, and therefore their puncture is not worth unless in vitro maturation (IVM) of oocytes is available? On the opposite side, is there a size threshold above which the follicle is not worth to be punctured, especially when oocyte pick-up (OPU) could be more challenging (for example in case of ovaries located behind the uterus or pelvic varicocele), with a higher risk of intra-abdominal bleeding?

Defining a universally accepted threshold of follicle size to predict the presence of good competent oocytes is difficult. Table 2 illustrates how the available data relating oocyte maturity and follicle size are fairly inconsistent. According to some authors, oocytes are frequently found in the metaphase II (MII) mature stage when retrieved from follicles above 16 mm mean diameter [17], whereas a higher proportion of immature, germinal vesicle (GV) or metaphase I (MI) oocytes is found in follicles below 12 mm [12, 18, 19]. Follicles above 22 mm often contain the so-called “post-mature” eggs, i.e. MII eggs that have undergone intra-follicular degenerative phenomena and are no more useful for fertilization [17]. Other studies put a 18 mm cut-off to retrieve with a better chance mature oocytes [19, 20]. Another study [21], tough showing a clear upward trend toward yielding mature oocytes from 16 mm follicles onward (approximately corresponding to a 2 ml volume), also reported that follicles from 11 to 15 mm sometimes can harbour mature oocytes. Other authors [22, 23] confirmed that follicles below 14 mm diameter seldom contain MII oocytes, both in normal and polycystic (PCO) ovaries. Overall there is evidence that follicles having a mean diameter between 16 and 22 mm are those with the highest likelihood of containing MII oocytes, but even small follicles can occasionally generate MII oocytes [24].

### May 2D-US imaging predict oocyte competence toward fertilization and embryo development?

Follicle number and size were reported to be independent predictors of likelihood of fertilization, morphological quality and number of embryos [25]. In a large retrospective study considering 2429 oocytes from 215 patients, follicular size above 16 mm at OPU was the best single indicator of oocyte fertilization potential, superior to the morphological characterization of the cumulus-oocyte complex [26]. Another study showed that fertilization and implantation rates with a leading follicle of 20 mm are higher than with a leading follicle of smaller size [27]. One of the largest studies, including 2934 oocytes, reported that oocytes with the best chance of fertilization are those from follicles above 18 mm, while the odd of retrieving MII oocytes and of fertilization progressively declines diminishing the follicle size [19]. Conversely, the rate of polispermy with conventional IVF was higher in follicles of smaller size, and embryos obtained from smaller follicles had a significantly higher fragmentation rate compared with those derived from eggs retrieved in bigger follicles [19]; these effects are likely due to a reduced oocyte competence, to either safeguard from more than one sperm getting in and to develop properly during first cleavage steps.

However, in none except two [17, 19] of these studies it was possible to identify a clear relationship between follicle size and morphological quality of the in vitro produced embryos. Possibly follicles between 16 and 22 mm contain oocytes of comparable quality, which finally leads to embryos of similar morphological score.

The next key question is: can follicular size be predictive of pregnancy? A retrospective study on 200 IVF cycles found that follicle size at OPU and follicular fluid volume were two of the only 4 parameters which resulted predictive of the take-home baby rate, among 53 examined [28]. Some authors reported that although oocytes from small follicles are sometimes found to be mature and yield fertilization, the resulting pregnancy rates are significantly lower compared to oocytes from follicles above 16 mm [18]. This finding suggests that even if meiotically mature, oocytes from smaller follicles could be defective in specific cellular events involving cytoskeletal organization and thus later affecting cytoplasmic maturation [29]. Contrasting observations, however, are reported: a large prospective study including 9933 follicles from 535 IVF cycles observed that oocytes from small follicles (12 mm or less) rarely get fertilized, but, once fertilized, they lead to apparently good quality embryos [30]. Similarly, when embryos obtained from oocytes originating from smaller follicles are transferred, the pregnancy rate is similar to the one observed with embryos derived from oocytes of larger follicles [31].

### Why data linking oocyte competence and size of the follicle are overall so inconsistent?

Conflicting results found in the published reports are likely due to several reasons including study design: some studies have a limited number of patients and/or are based on retrospective observations. There are also merely technical reasons: the evaluation of oocyte maturity at the stereomicroscope, usually performed soon after oocyte retrieval, may be misleading as some oocytes that immediately appear as MII could still be in the telophase of the first meiotic division. This “morphological artifact” may have induced some authors to overestimate the rate of maturity when oocytes retrieved from small follicles were considered. Secondly, often different methods to determine follicular dimensions are adopted: either the follicular fluid volume collected at OPU is used to estimate follicular volume, or follicle dimensions are measured by US just prior to oocyte puncture. The methodology used in different studies is important to know, since follicles have a median growth of approximately 2 mm from the day of ovulation triggering to the day of OPU [20].

Though any of the above-mentioned studies reported separated data according to COH protocols, also the type of ovarian stimulation is likely to affect the genetic and/or biochemical intra-follicular environment. As a consequence, different medications and/or protocols could influence the size at which follicles contain competent oocytes. For example, when clomiphene citrate (CC) is employed in COH, alone [32] or added to gonadotropins [33], oocytes from follicles <18/<20 mm show significantly lower fertilization potential than oocytes derived from larger follicles. Possibly when the pituitary secretion of endogenous gonadotropins is stimulated (with the use of clomiphene citrate), the follicle size at which the eggs reach optimal competence is higher than observed when a direct ovarian stimulation with exogenous gonadotropins is used. It was also reported that when GnRH-antagonists are used (and thus the first week of follicle growth occurs without pituitary block, with the contribute of endogenous gonadotropins), oocyte maturation is obtained at a lower follicle size than when a GnRH-agonist is given in the classical “long” protocol (and the pituitary block precedes COH) [34]. Another possible bias among studies could be the fertilization type involved. Some authors reported separately results from IVF and ICSI cycles [17–19] and usually ICSI seems to be less affected from different follicle sizes.

Interestingly, none of the studies reported any correlation between IVF outcomes, follicular diameters and age of the patients enrolled. Overall, in many studies the average age is approximately 34–35 years old, but with a 4 to 5 years of standard deviation: this extends the age of the patient population to a large range, from before thirties until early forties when IVF outcomes could be very different. Further, any study correlate follicular size to days of stimulation needed to reach maturity, while this parameter could be easily recorded and maybe correlate to female age and recovery rate of MII oocytes.

Even intrinsic defects of 2D-US technique may contribute to generate contrasting data: for example lack of standardization of US measurement among members of the team or the use of different ultrasound scanners and settings may lead to different results. The manual 2D-US monitoring of follicular growth, in fact, has important limitations when many follicles are simultaneously growing in each ovary. 2D-US imaging, in fact, makes assumption that an object has a regular shape and uses the measurement of two axis as surrogates to estimate its true size: this may be inaccurate for follicles having uneven and irregular shapes, like those developing during COH. Furthermore, accurate follicles measurement could be even more difficult to perform in the presence of anatomical alteration. Indeed, endometriomas are frequently detected in an infertile population, and it was reported that both ovarian-volume measurement and antral follicle count lose accuracy in the presence of ovarian cysts as well as in the presence of a corpus luteum [35, 36].

Finally, measuring the mean diameter of multiple follicles is time-consuming, and may result in crowded waiting rooms and frustrated patients; this forces the doctor to work quickly and, probably, with less precision. The final result is an intra- and inter-observer variation that has been estimated to be around 20% when several follicles are contemporaneously present inside the ovaries [37].

### 3D-US study of follicle volume and the prediction of oocyte competence

A new opportunity to get useful insights about our topic could be presently offered by the 3D-US scanning. Currently 3D-US may be performed by manual measurement of follicle circumference through Virtual Organ Computer-Aided Analysis (VOCAL) or by an automated US application, the Sonography-based Automated Volume Calculation (SonoAVC) [38, 39].

Manual measurement and SonoAVC correlate quite well in the assessment of follicle morphology [40, 41], but SonoAVC has a higher intra- and inter-observer reproducibility than VOCAL [42], and can also be used interchangeably together with conventional 2D measurement [43–45].

SonoAVC automatically identifies the boundaries of follicles and provides an estimate of their largest diameters in three orthogonal planes [38]. This technology implies 3D image manipulation and post-processing [46]. Each individual follicle is identified on the screen with a specific color and later shown together with all other follicles, with dimensions and relative sizes calculated using the relaxed sphere technique. The volume calculation is based on *voxel* count within the identified hyper-echoic structure and finally represents a true measure of follicular volume regardless of the regularity of its shape [47, 48]. This is a large improvement versus conventional 2D-US: in fact, when the measured follicle is other than spherical, evaluation of the mean follicular diameter risks to be an overestimate and it is a matter of fact that ovaries under COH predominantly harbor ellipsoid follicles [21].

Both VOCAL and SonoAVC provide an accurate way to measure ovarian follicle volume: a 95% correspondence between their measurement and the actual follicle volume (obtained by measuring the volume of the aspirated follicular fluid) was reported [42, 47, 48]. Deutch [40], showed <0.02 ml error comparing spheres of known volume with a hyper-echoic matrix using an ultrasound phantom. Rousian [49], who used spheres having larger volume than the previous study, showed that the SonoAVC system slightly underestimates the volume (by a mean difference of -0.63 ml).

The advantage of SonoAVC over 2D-US may be the possibility of getting follicle measurement automatically - and consequently more quickly - and with a very low inter-observer variability, overcoming the lack of standardization in US technique that represents an important limitation of 2D-US. SonoAVC also allows to virtually repeat US examination in the absence of the patient: this appears to be useful both for training resident doctors and to be used as a method of quality control and standardization of the technique.

Some limitations are also present using 3D-US methods: the ultrasound machines are highly costly and this could limit affordability for smaller infertility centers; furthermore 3D machine still need to rely on 2D-scan when good quality images cannot be recorded [50].

To date, only few studies evaluated the use of SonoAVC for monitoring follicular growth in stimulated ovaries. A very good correspondence between 2D and SonoAVC measurements of follicle size was reported, and SonoAVC was found to significantly reduce the time needed to perform US examination [38, 40, 43, 48, 50, 51]. Of notice is that the reduced time reported for SonoAVC is the comprehensive time for both volume acquisition, post processing when needed and data analysis. Unfortunately, none of the studies performed to date was aimed at testing how the 3D-US technique could elicit any improvement in IVF success rate [42, 52]; in fact, in these studies, hCG ovulation trigger injection was administered as usual (according to the mean diameter of the leading follicle) and not looking at its 3D-measured volume. The only study comparing results between women monitored with either 2D-US and SonoAVC reported similar number of mature oocytes retrieved, fertilization and clinical pregnancy rates [51].

Anytime a new tool is introduced, we first collect conflicting reports. In the past, for example, when vascularity assessment was performed, only some authors correlated IVF outcome to power Doppler parameters [25, 53, 54]; thus suggesting the need to standardize the technique and to integrate it with standard 2D-US, before introduction in the clinical practice. Similarly, though reassuring reports on 3D-US previously reported, there is still the need to perform new studies on follicular volume calculation as a new criterion to decide when ovulation trigger should be performed.

## Conclusions

The morphological study of the follicle size using 2D-US equipment is routinely performed in IVF Units worldwide, and its tolerability, real time results renders it, in theory, the ideal tool to get useful indications about oocyte competence. Unfortunately, the available evidence fails to clearly indicate a size range within which follicles contain oocytes of good quality, and gives just approximate ideas. Mature, competent oocytes are more likely to derive from follicles that have a mean diameter between 16 and 22 mm when their final maturation is triggered. Fertilizable oocytes may sometimes be retrieved from smaller follicles both in women with normal or polycystic ovaries, but the derived embryos are suspected to have a reduced implantation potential than those originated from eggs contained in follicles within the above mentioned size range.

The available data about oocyte competence and follicle size, however, are quite inconsistent because of the small size and retrospective nature of most studies, for possible “biological artifacts” in assessing oocyte maturity, as well as for the intrinsic limitations of the 2D-US technique. As a consequence, at present we cannot make a clear distinction on when to trigger ovulation or wait, and on which follicle to aspirate or not on the basis of its US characteristics.

Better knowledge in this area could be helpful to optimize IVF outcome by refining COH protocols and obtain high quality oocytes. It is questionable but possible that prospective studies performed using 3D automated US machines, either alone or with Doppler evaluation, will be able to standardize follicle assessment and reduce the intra- and inter-observer variability.
